# Strong founder effect of p.P240L in *CDH23* in Koreans and its significant contribution to severe-to-profound nonsyndromic hearing loss in a Korean pediatric population

**DOI:** 10.1186/s12967-015-0624-8

**Published:** 2015-08-13

**Authors:** So Young Kim, Ah Reum Kim, Nayoung K D Kim, Min Young Kim, Eun-Hee Jeon, Bong Jik Kim, Young Eun Han, Mun Young Chang, Woong-Yang Park, Byung Yoon Choi

**Affiliations:** Department of Otorhinolaryngology-Head and Neck Surgery, Seoul National University Hospital, Seoul National University College of Medicine, Seoul, Korea; Samsung Genome Institute, Samsung Medical Center, Seoul, Korea; Department of Otorhinolaryngology-Head and Neck Surgery, Seoul National University Bundang Hospital, Seoul National University College of Medicine, 300 Gumi-dong, Bundang-gu, Seongnam, 463-707 Korea; Department of Otorhinolaryngology-Head and Neck Surgery, Dankook University Hospital, Cheonan, Korea; Sensory Organ Research Institute, Seoul National University Medical Research Center, Seoul, Korea; Department of Molecular Cell Biology, School of Medicine, Sungkyunkwan University, Seoul, Korea

**Keywords:** CDH23, p.P240L, Founder effect

## Abstract

**Background:**

Despite the prevalence of *CDH23* mutations in East Asians, its large size hinders investigation. The pathologic mutation p.P240L in *CDH23* is common in East Asians. However, whether this mutation represents a common founder or a mutational hot spot is unclear. The prevalence of *CDH23* mutations with prelingual severe-to-profound sporadic or autosomal recessive sensorineural hearing loss (arSNHL) is unknown in Koreans.

**Methods:**

From September 2010 to October 2014, children with severe-to-profound sporadic or arSNHL without phenotypic markers, and their families, were tested for mutations in connexins *GJB2*, *GJB6* and *GJB3*. Sanger sequencing of *CDH23* p.P240L was performed on connexin-negative samples without enlarged vestibular aqueducts (EVA), followed by targeted resequencing of 129 deafness genes, including *CDH23,* unless p.P240L homozygotes were detected in the first screening. Four p.P240L-allele-linked STR markers were genotyped in 40 normal-hearing control subjects, and the p.P240L carriers in the hearing-impaired cohort, to identify the haplotypes.

**Results:**

Four (3.1 %) of 128 children carried two *CDH23* mutant alleles, and *SLC26A4* and *GJB2* accounted for 18.0 and 17.2 %, respectively. All four children showed profound nonsyndromic SNHL with minimal residual hearing. Interestingly, all had at least one p.P240L mutant allele. Analysis of p.P240L-linked STR markers in these children and other postlingual hearing-impaired adults carrying p.P240L revealed that p.P240L was mainly carried on a single haplotype.

**Conclusions:**

p.P240L contributed significantly to Korean pediatric severe arSNHL with a strong founder effect, with implications for future phylogenetic studies. Screening for p.P240L as a first step in *GJB2*-negative arSNHL Koreans without EVA is recommended.

**Electronic supplementary material:**

The online version of this article (doi:10.1186/s12967-015-0624-8) contains supplementary material, which is available to authorized users.

## Background

Many loci can cause genetic sensorineural hearing loss (SNHL), which accounts for more than 50 % of SNHL [1]. New molecular genetic techniques, such as whole-exome sequencing [2] and targeted resequencing (TRS) [3], have enabled more in-depth understanding of the molecular etiologies of SNHL. These next-generation sequencing (NGS) technologies have assisted in screening of large deafness genes.

*CDH23* (NM_022124) is a prime example as this gene consists of 69 exons, 11,073 bases, and is located on chromosome 10q21–q22 [4–7]. It encodes cadherin 23, consisting of 3,354 amino acids and forms 27 extracellular domains (EC), a single transmembrane domain and a short cytoplasmic domain. This protein is reported to contribute to tip link formation by interacting with protocadherin 15 which is encoded by *PCDH15* (NM_001142771). It thereby maintains the structural integrity of the tip link and the mechanoelectrical transducer currents of hair cells [8, 9]. Mutations in *CDH23* lead to two types of phenotypic disorder or nonsense, splice-site, frameshift mutations, which are assumed to severely disrupt the functions of cadherin 23, usually associated with Usher syndrome type 1D (USH1D; MIM601067) [5]. In contrast, hypo-functional missense mutations result in nonsyndromic hearing loss, preserving retinal and vestibular functions (DFNB12; MIM601386) [5, 10]. When these hypofunctional (DFNB12 allele) and nonfunctional mutations (USH1D allele) are in *trans* configuration to each other, it is presumed that the residual function of the DFNB12 allele preserves vision and the vestibular function, and impacts the function of only hair cells in the inner ear, resulting in the DFNB12 phenotype [11, 12]. To date, more than 50 mutations have been identified in USH1D patients, and over 24 mutations in DFNB12 patients [13].

The advent of NGS rapidly led to identification of *CDH23* as a cause of deafness, especially in East Asians. Indeed, *CDH23* mutations contributed significantly to SNHL, with genetic loads that differed according to the ethnicity of the population [10, 14–16]. In East Asians, *CDH23* mutations were the third-most frequent after *GJB*2 and *SLC26A4*. They accounted for 3.7 % of total hearing loss patients and 5.7 % of autosomal recessive SNHL (arSNHL) patients in Japan [13]. Recently, Park et al. [17] reported an association between *CDH23* mutations in hearing loss in 3 (3.22 %) of 93 Korean cochlear implantees with varying degrees of onset, and with diverse inheritance patterns [17]. Another small Korean cohort also had *CDH23* mutations (2/13 [15 %]) resulting in autosomal recessive hearing loss with negative *GJB2* and *SLC26A4* mutations [18]. Most of the patients had progressive, moderate-to-profound, high-tone hearing loss with some residual hearing in the lower frequencies, without vestibular or retinal dysfunction [12, 19].

Rigorous and costly bioinformatic analyses are needed to screen for these mutations, and despite significant reductions in the cost of some NGS techniques, routine clinical screening for *CDH23* remains expensive and not cost-effective. Nevertheless, p.P240L, a predominant *CDH23* mutation in Japanese and Koreans, is of interest as it accounts for ~43.3 % and >50 % of total *CDH23* mutations, respectively [13, 18, 19]. The presence of a predominant allele would facilitate identification of DFNB12 subjects. However, whether p.P240L is a hotspot mutation, a founder mutation, or both, remains to be determined.

In this study, the relative genetic contribution of the *CDH23* mutation to nonsyndromic arSNHL was estimated in a Korean pediatric hearing loss cohort, and the genetic load of *GJB2* and *SLC26A4* was documented. Moreover, whether p.P240L, the most common *CDH23* mutation in Koreans, represented a common disease-associated founder mutation was investigated. The clinical characteristics (onset of hearing loss, progression, audiologic evaluation) of the hearing-impaired subjects carrying *CDH23* mutations were also documented.

## Methods

### Ethical considerations

This study was approved by the Institutional Review Boards (IRBs) at the Seoul National University Bundang Hospital (SNUBH) (IRB-B-1007-105-402) and the Seoul National University Hospital (SNUH) (IRBY-H-0905-041-281). Written informed consent was obtained from all participating subjects. In case of the children, written informed consent was obtained from their parents or guardians on their behalf.

### Study participants and audiometric evaluation

A total of 438 subjects who visited hereditary hearing loss clinics of tertiary referral centers: (SNUH and SNUBH) for their SNHL from September 2010 to October 2014 were initially enrolled in this study. Clinical evaluations were performed in this cohort, including gender, date of birth, medical history, physical examination, pure tone audiometry and when available, imaging studies (temporal bone computed tomography or magnetic resonance imaging).

All of the enrolled subjects underwent an audiologic evaluation. The pure-tone thresholds were recorded at 0.25, 0.5, 1, 2, 4 and 8 kHz. However, some infants could be recorded at only limited frequencies due to poor cooperation. The hearing threshold was calculated by averaging the thresholds of 0.5, 1, 2 and 4 kHz, and was classified as subtle (16–25 dB), mild (26–40 dB), moderate (41–70 dB), severe (71–95 dB), or profound (>95 dB).

**Fig. 1:**
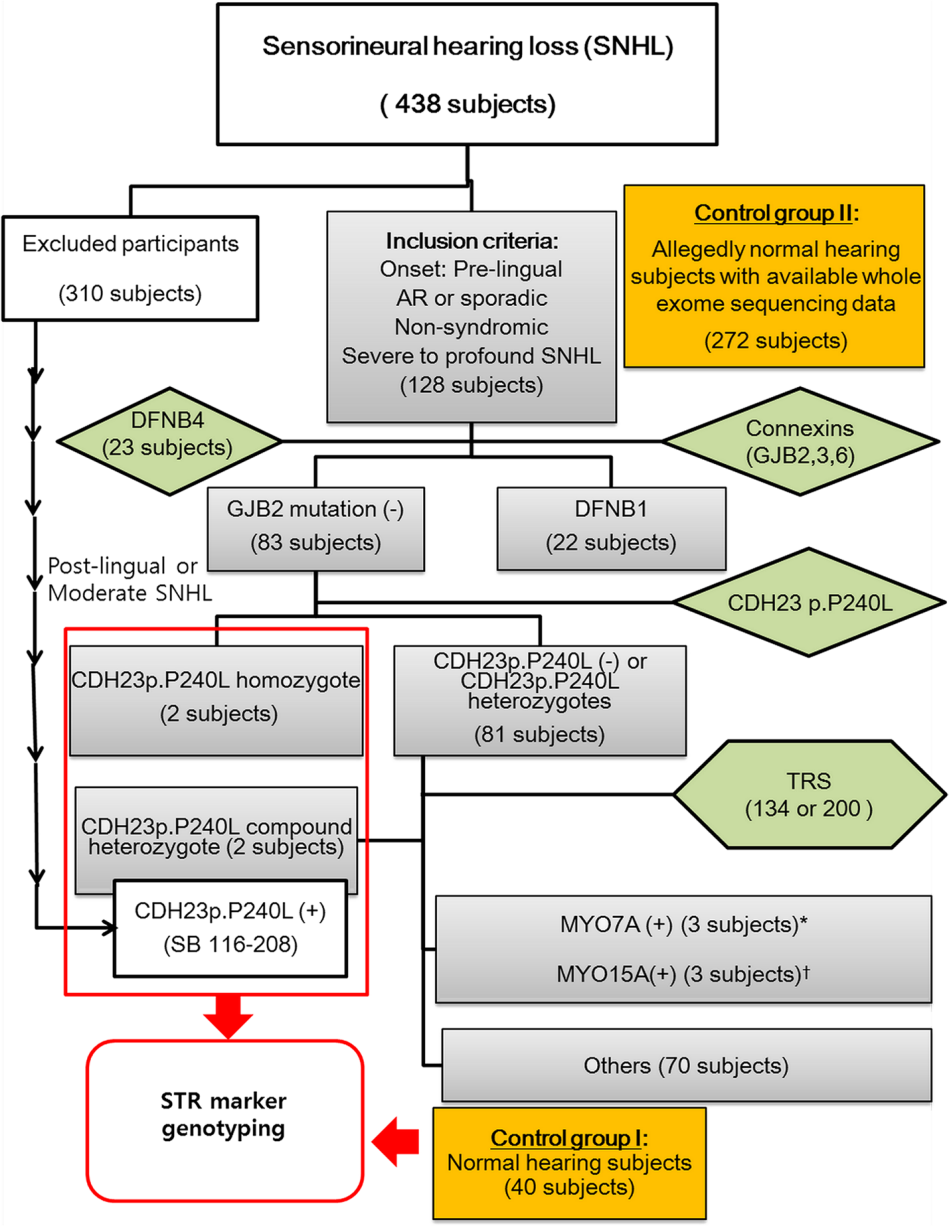
A diagnostic flow chart of the enrolled subjects. Of a total of 438 subjects with various onset and degree of sensorineural hearing loss (SNHL) diagnosed from 2010 to 2014 at SNUH or SNUBH, 128 with the prelingual onset of a severe-to-profound degree of hearing loss, and with an autosomal recessive (AR) or sporadic hereditary pattern were included in this study. The 310 subjects either with pre-lingual mild to moderate degree SNHL or with post-lingual SNHL subjects are excluded from the present study. *SLC26A4* and/or *GJB2* were sequenced, and the p.P240L of *CDH23* was screened in subjects without *SLC26A4* or *GJB2* mutations. One of the subjects (SB166-208) with post-lingual SNHL identified to carry p.P240L of *CDH23* is included in the STR marker genotyping analysis. Next, targeted resequencing (TRS) of the 80˗204 known deafness genes was performed. *Asterisks* indicates MYO15A compound heterozygotes; SB77-133, SH10-28, SHJ23, *dagger* indicates MYO7A compound heterozygotes; SH156-272, SH91-202, SB71-123. Control group I (272 subjects) was assigned for p.P240L mutation screening and control group II (40 subjects) was assigned for STR genotyping. All control groups showed normal hearing.

Based on the results of the above clinical and audiologic evaluations, pediatric subjects with prelingual onset, severe-to-profound SNHL segregating either in a sporadic or an autosomal recessive fashion were selected for this study. Individuals with unilateral or phenotypic markers in the inner ear structure, such as an incomplete partition type III, were excluded. However, subjects with an enlarged vestibular aqueduct (EVA) were included to compare the *CDH23* mutation frequency with those of mutations in *SLC26A4* and other deafness genes in the same cohort (Fig. 1). Consequently, 128 unrelated children with seemingly nonsyndromic severe-to-profound sporadic, or arSNHL without other notable abnormality were finally included in this study. All recruited subjects were Korean.

We recruited two control groups: the control group I included randomly chosen 40 Korean adult subjects with pure tone audiograms indicating normal hearing thresholds and the control group II comprised independent 272 adult subjects with available whole exome sequencing data and with allegedly normal hearing. In this control group II, whole exome sequencing was performed prior to this study to make a molecular diagnosis of diseases irrelevant to SNHL and their hearing status was evaluated by the interview. The control group I and II was recruited for the analysis on the haplotypes around p.P240 of *CDH23* and calculation of the normal carrier frequency of p.P240L, respectively.

### DNA samples and mutation analysis

Molecular genetic studies were conducted in 128 probands and their family members. Genomic DNA was extracted from peripheral blood using standard protocols (Gentra Puregene Blood Kit, Qiagen, cat. 158389; Venlo, Limburg, Netherlands). The nonsyndromic prelingual arSNHL subjects with an enlarged vestibular aqueduct (EVA) underwent direct PCR-based Sanger sequencing of *SLC26A4*. Sanger sequencing of *GJB2*, 3 and 6 was performed on subjects without noticeable phenotypic markers, as described previously [20] (Fig. 1).

Non-EVA samples that were also negative for mutations in connexins *GJB*2, *GJB3*, and *GJB6* were subject to Sanger sequencing of p.P240L, which was the most frequent mutant allele of *CDH23* in Korean cochlear implantees, as reported in our previous study [17] (Fig. 1). The primer sequence for Sanger sequencing to screen p.P240L of *CDH23* was *CDH23*-8F(5′-CCACCATCACTCAACCTAAG-3′) and *CDH23*-8R (5′-ACTAACAGGGCCCATCCCTA-3). Next, targeted resequencing of 80, 129 (in collaboration with Otogenetics (Norcross, GA, USA) or 200 known deafness genes [17] including *CDH23,* was performed on the remaining non-EVA samples without a conclusive molecular diagnosis after sequencing of *GJB2*, *GJB3*, and *GJB6*, as described previously [17, 21], with the exception of those samples in which homozygous p.P240L mutations were detected at the screening of p.P240L by Sanger sequencing (Fig. 1). An additional file shows the supplementary lists of the genes included in the three targeted panels (see Additional file 1). This process identified DFNB12 children carrying two probable pathogenic *CDH23* variants in a *trans* configuration.

### STR marker genotyping

**Fig. 2:**
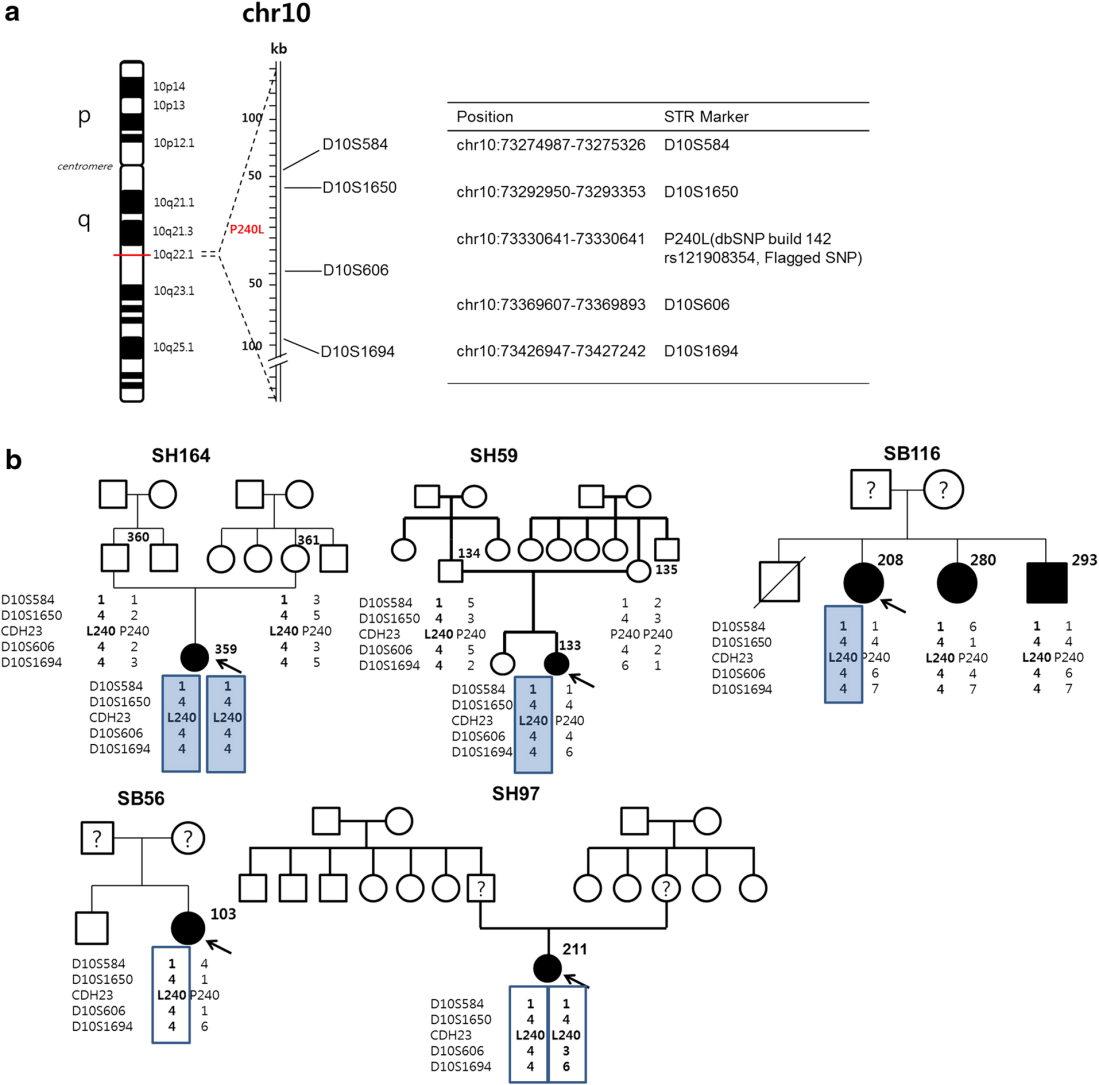
**a** Location of p.P240L of *CDH23* and 4 STR markers linked to the p.P240L mutation on chromosome 10q22.1. Four STR makers were located in the flanking regions of the *CDH23* gene. The D10S1650 and D10S1694 were previously reported STR markers to be hypervariable and informative. **b** Pedigrees showing the segregation of the p.P240L mutation and the four STR marker haplotypes. Haplotypes for STR markers linked to p.P240L from five probands (*black arrow*) are indicated in *bold* in either *open* or *shaded boxes*). A single haplotype for four STR markers (D10S584, D10S1650, D10S606, and D10S1694) is definitely shared by four alleles linked to p.P240L (*shaded box*) including one allele from a postlingual-hearing-loss adult (SB116-208), indicative of a very strong founder allele in Koreans. The single common haplotype can be potentially assigned to two of three alleles (*open box*) carrying p.P240L from two subjects (SB56-103 and SH97-211. Definite haplotypes could be assigned based on information from parental samples or affected siblings in SH164-359, SH59-133, and SB116-208. Despite a lack of parental or sibling samples, a most-likely probable haplotype could be assigned based on the haplotypes of other subjects carrying p.P240L in SB56-103 and SH97-211.

The haplotype of the p.P240L allele of *CDH23* in Koreans was determined to ascertain whether the mutant allele was a common founder. For this purpose, we chose four STR markers flanking the *CDH23* gene (Fig. 2a) which included two markers (D10S1650 and D10S1694) previously reported to be hypervariable and informative [5, 11]. Primers for four STR markers flanking the *CDH23* gene were generated based on the UCSC Genome Browser as described [21]. The sequences of the microsatellite markers were: D10S584 (5′-TCAATGGGAATGGATACC-3′) (5′-GCAGATCCGAACATGG-3′), D10S1650 (5′-GAAGCCTGTGGTCTAATGAG-3′) (5′-TTCTGGCCTCTGCAGC-3′), D10S606 (5′-TTTGAACCTGGGAGACG-3′) (5′-CATGGACATTCTGCTGC-3′), D10S1694 (5′-CCTGTCTGGCCCAGGTA-3′) (5′-AGTAGGGGTGCTGCTTGA-3′) [5, 11]. Loci were amplified using AmpliTaq DNA polymerase (Invitrogen Life Technologies, Carlsbad, CA, USA) in a Perkin Elmer 9700 thermal cycler (Perkin Elmer, Waltham, MA, USA). The genotypes of these loci on 10 chromosomes were defined using an AmpliTaq gold (Applied Biosystems, Waltham, MA, USA) touchdown protocol under the following conditions: 10 min at 95 °C; 10 cycles of 30 s at 94 °C, 30 s at 65 °C, and 30 s at 72 °C; and a further 20 cycles under the same conditions but with an annealing temperature lowered from 0.5 to 55 °C, 15 cycles of annealing at 55 °C, and a 72 °C final extension for 10 min. STR marker genotyping was performed as described previously [22].

One multiplex family (SB116) with co-segregating post-lingual onset of SNHL and a heterozygous p.P240L mutant allele of *CDH23* was also included in this analysis. STR marker analyses were conducted in 11 subjects from five SNHL families (four prelingual and one post-lingual) in total, segregating p.P240L of *CDH23*, and also in 40 adult Korean subjects with documented normal hearing thresholds.

### Statistical analysis

The allele frequency of p.P240L was compared between prelingually onset, severe-to-profound sporadic/autosomal recessive SNHL in a pediatric population and 272 normal hearing controls (control group II). Differences in STR genotypes and the distribution of haplotypes between mutant chromosomes carrying p.P240L and 80 wild-type Korean chromosomes from 40 normal-hearing subjects (control group I) were analyzed by Fisher’s exact test. One postlingual adult DFNB12 subject (SB116-208) was additionally included for the STR genotyping, while the subject was excluded for calculation of the allele frequency of p.P240L among pediatric prelingual severe to profound SNHL. Contributions of some genotypes were compared among subjects, but not between chromosomes, due to the lack of parental samples, as described previously [17, 23]. Especially, haplotypes of normal controls were constructed in a conservative way that was toward minimizing the statistical difference of haplotypes between p.P240L carriers with SNHL and normal controls.

Statistical analyses were performed using SPSS 18.0 (SPSS Inc., Chicago, USA). The level of statistical significance was defined as a *p* value of <0.05.

## Results

### The contribution of mutations in *CDH23* to profound nonsyndromic arSNHL in Korean children

**Fig. 3:**
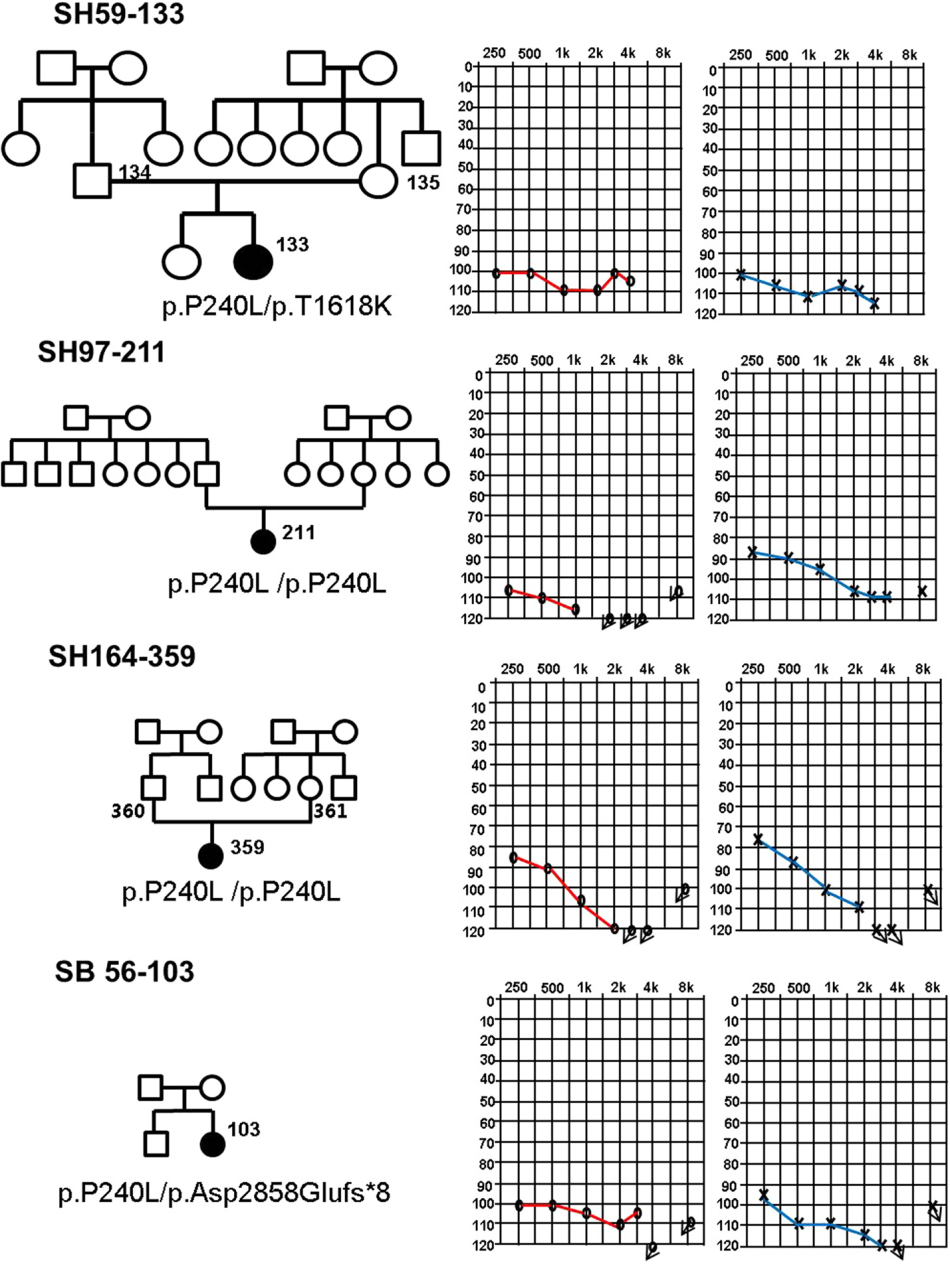
Pedigrees and audiograms of the subjects carrying the *CDH23* mutation. All four carriers of p.P240L in *CDH23* showed severe-to-profound autosomal recessive prelingual SNHL (arSNHL) with minimal residual hearing.

Among 128 children with prelingual onset, nonsyndromic, sporadic or autosomal recessive, severe-to-profound SNHL, 22 (17.2 %) had two mutant alleles of *GJB2* (DFNB1) and 23 (18.0 %) carried two mutant alleles of *SLC26A4* (DFNB4). The detection frequency of *CDH23* mutations (SH 59-133 [17], SH 164-359 [17], SB56-103 and SH 97-211) in our pediatric cohort was 4/128 (3.1 %), which was slightly higher than the 3/128 (2.3 %) and 3/128 (2.3 %) of *MYO15A* and *MYO7A*, respectively, in the same cohort. Considering the fact that we did not screen *CDH23* from 22 subjects with two mutant alleles of *GJB2*, the actual frequency of *CDH23* might be even slightly high than this figure. Our result showed that *CDH23* was one of the leading causes of prelingual-onset profound nonsyndromic arSNHL in children and was a component of the suite of genes that includes *SLC26A4* and *GJB2,* in the Korean pediatric cohort (Fig. 1). The mean age at detection of hearing loss was 2.25 years, with a range of 1–4 years. All four subjects with *CDH2*3 mutations showed a similar phenotype and prelingual profound hearing loss with minimal residual hearing (Fig. 3). None of the four *CDH23* carriers exhibited symptoms of nyctalopia, and ophthalmologic examinations revealed no definite abnormalities.

### The p.P240L mutant allele of *CDH23* predominated in the pediatric cohort

Interestingly, all four subjects carried p.P240L as either a homozygote or compound heterozygotes, suggesting that this mutant allele was either a strong common founder or a mutational hotspot in this population (Table 1) (Fig. 3).

**Table 1:** Mutations of the *CDH23* gene identified in this study

Patient	Sex/age	Classification	Nucleotide change^a^	Amino acid change^b^	Inheritance pattern
SH59-133	F/3	Children	c.C719T; c.C4853A	p.P240L; p.T1618K	Compound heterozygote
SH97-211	F/1	Children	c.C719T	p.P240L	Homozygote
SH164-359	F/1	Children	c.C719T	p.P240L	Homozygote
SB56-103	F/4	Children	c.C719T; c.8574delC	p.P240L;p.Asp2858Glu*fs*X8	Compound heterozygote

^a^NM_022124.

^b^NP_071407.

To ascertain whether p.P240L was a causative variant in these subjects, the carrier frequency of the p.P240L allele was calculated among 272 adult controls with allegedly normal hearing thresholds (control group II). None of the normal control subjects carried the mutant allele, which further confirmed its pathogenic potential (6/256 vs 0/544, p = .001, by Fischer’s exact test) (Table 2).

**Table 2:** Prevalence of *CDH23* mutations in East Asian

Characteristics	Study1	Study2	Study3,4
Ethnicity	Korean	Korean	Japan
Onset of hearing loss	Prelingual	Early onset	Pre & postlingual
Inheritance	AR, or sporadic	AR	AR, or sporadic
Degree of hearing loss	Severe-to-profound	Severe-to-profound	Various
Syndromic/nonsyndromic	Nonsyndromic	Nonsyndromic No *GJB2* and *SLC26A4* mutations	Nonsyndromic
Frequency of *CDH23* mutations	3.13 (4/128) 4.82 (4/83)^a^	15.38 (2/13)	3.72 (52/1396)^b^ (33, postlingual, 19, prelingual) 1.6 (23/1396)^c^ 2.5 (23/919)^d^
Frequency of p.P240L alleles			
Affected	2.34 (6/256)	7.69 (2/26)	1.61 (45/2792)
Control	0 (0/544)	0.12 (2/1636)	0.26 (1/384)
Affected (in *CDH23* mutations)	85.71 (6/8)	50.00 (2/4)	60.01 (45/2792)
References	This study	[18]	[19] [24]

*AR* autosomal recessive inheritance.

^a^The prevalence of *CDH23* mutations when excluding the subjects with *GJB2* or *SLC26A4* mutations.

^b^The prevalence of *CDH23* mutations with 10 homozygote, 13 compound heterozygote, excluding single heterozygote cases, 29 heterozygote.

^c^The prevalence of *CDH23* mutations with 10 homozygote, 13 compound heterozygote.

^d^The prevalence of *CDH23* mutations in autosomal recessive subjects, excluding sporadic cases.

### The rate of concordance in STR markers linked to the p.P240L mutation

p.P240L was assumed to act as a common founder mutation or a mutational hot spot. To ascertain whether p.P240L was a common founder, p.P240L-linked haplotypes of four STR markers (D10S584, D10S1650, D10S606, and D10S1694) were constructed and analyzed in five unrelated probands from five families (four prelingual [SH 59, SH164, SB56, and SH97] and one postlingual [SB116]), and in 40 adult Korean normal-hearing subjects (control group I) (Table 3; Fig. 2b). For two pediatric probands (SH164-359 and SH59-133) and one adult proband (SB116-208), haplotypes could definitely be assigned, based on information from the parents and affected siblings (Fig. 2b). For the remaining two pediatric probands (SH56-103 and SH97-211), precise haplotypes could not be assigned due to a lack of parental or sibling samples. Instead, their most likely probable haplotype was inferred from the other p.P240L-carrying subjects in our cohort (Fig. 2b). In detail, our three p.P240L-carrying probands (SH164-359, SH59-133, and SB116-208) with definitive STR haplotypes shared a common single haplotype (shaded box in Fig. 2b) in their all four alleles linked to p.P240L. Based on this, the other two subjects (SB56-103 and SH97-211) were also assumed to have this common haplotype (Fig. 2b). However, these two subjects (SB56-103 and SH97-211) might have a different haplotype linked to p.P240L against our assumption. Nonetheless, at least four (57.1 %) (shaded box in Fig. 2b) (two alleles from SH164-359, and each allele from SH59-133 and SB116-208) of the seven p.P240L-linked alleles (shaded or open box in Fig. 2b) showed identical haplotypes (Fig. 2b). In contrast, the normal controls showed diverse haplotypes for these STR markers, and the identical haplotype present in the p.P240L carriers could not be assigned to any of the normal controls (0/80) (Table 3). Consequently, p.P240L was significantly associated with a single haplotype in our cohort even under a conservative setting where SH56-103 and SH97-211 were assumed to have a different haplotype (57.1 % [4/7] vs. 0 % [0/80], p < .0001 by Fisher’s exact test). However, the identical haplotype could be inferred also in two subjects (SH56-103 and SH97-211) without any additional samples from their families by assuming the most likely haplotype, making the proportion of shared identical haplotypes 85.7 % (6/7). Conversely, when only three STR markers were considered (D10S584, D10S1650, and D10S606), seven alleles from the normal controls (8.75 %, 7/80) could potentially be matched to an identical haplotype that was strongly associated with p.P240L. Even under this assumption, a highly significant statistical association was detected between p.P240L and a single haplotype (p < .001 by Fisher’s exact test) (Table 3).

**Table 3:** Chromosome 10q22.1 STR haplotypes linked to p.P240L of *CDH23*

Haplotype	STR marker genotype				Korean chromosome	
	D10S584	D10S1650	D10S606	D10S1694	P240L (n = 7)	Control (n = 80)
1	184	138	238	149	6* (4)**	0**
2	184	138	236	155	1	0
3	182	136	230	139	0	4
4	184	138	238	147	0	2*
5	184	138	238	145	0	2*
6	184	138	230	139	0	2
7	184	138	234	141	0	2
8	184	134	238	147	0	2
9	184	136	238	139	0	2
10	184	134	234	147	0	2
11	184	130	230	139	0	2
12	190	134	234	147	0	2
13	184	138	238	139	0	1*
14	184	138	238	141	0	1*
15	184	138	238	153	0	1*
16	184	138	240	149	0	1
17	184	138	232	145	0	1
18	184	138	236	147	0	1
19	184	138	236	145	0	1
20–69	–	–	–	–	0	51

* Meiotic phase and chromosomes in SH56-103 and SH97-211(SHJ71) and in most of the normal hearing controls could not be decisively assigned due to lack of parental DNA samples: In these cases, genotype contributions were compared among subjects, not chromosomes as described [23] (p > 0.001 by Fisher exact test). Haplotypes of normal controls were constructed in a conservative way where the haplotype closest to the haplotype 1 (see above) was preferentially assumed from STR genotyping data. **The number in parenthesis indicates a genotype contribution among chromosomes confirmed through checking parental samples (p > 0.001 by Fisher exact test).

## Discussion

The prevalence of recessive *CDH23* mutations in our pediatric cohort with severe-to-profound nonsyndromic arSNHL was 3.1 %. This was in agreement with findings for *CDH23* variants in other studies on East Asian populations, the prevalences of which ranged from 1.6 to 15.4 %, using cohorts with nonsyndromic arSNHL not necessarily limited to prelingual pediatric deaf subjects (Table 2) [18, 19, 24, 25]. The wide range in the frequency from 1.6 to 15.4 % may be attributed to ethnicity differences among East Asians. Alternatively, an intrinsic difference in the study cohort, such as cohort size or onset age of SNHL, could also account for these differences. In this study, the cohort consisted exclusively of prelingual nonsyndromic arSNHL pediatric subjects. Therefore, significantly higher frequency of *CDH23* mutations (15.4 vs 3.1 %) obtained from a different Korean cohort with nonsyndromic arSNHL not limited to prelingual SNHL cases [18] (Table 2) may suggest *CDH23* mutations also significantly contribute to adult-onset postlingual arSNHL in Koreans. Indeed, in contrast to the p.P240L mutation, which presented mostly as prelingual severe-to-profound hearing loss, certain *CDH23* mutations, such as p.R2029W and p.T1368M, were reported to be associated with postlingual onset of moderate hearing loss, and most *CDH23*-affected subjects showed progressive hearing loss [13]. These results warrant further investigation in a separate study.

*CDH23* mutations were the third-most prevalent molecular etiology in the pediatric prelingual arSNHL cohort, after *SLC26A4* and *GJB2,* which accounted for 18.0 and 17.2 %, respectively. A mutation of p.P240L in *CDH23* was detected in all four DFNB12 subjects, as either a compound heterozygote or a homozygote, in the Korean cohort. Screening of p.P240L alone significantly facilitated detection of DFNB12 or DFNB12 candidates in Korean subjects, despite the large size of this gene. Given the prevalence of p.P240L, incorporation of a test for p.P240L in *CDH23* as a next step in *GJB2* sequencing in non-EVA subjects would be cost-effective (Fig. 1).

Indeed, several studies have reported the predominance of the p.P240L mutation in Japanese and Korean populations affected by the prelingual or postlingual arSNHL [18, 19, 24]. In line with these studies, our results showed that 85.7 % (6/8) of the alleles of the *CDH23* mutation carriers in the Korean pediatric population had p.P240L (Table 2). Based on the frequency of this variant, we hypothesized that p.P240L of *CDH23* had a founder effect in East Asians. Based on our haplotype analyses, p.P240L was mainly carried on a single common haplotype and the common haplotype linked to p.P240L was not detected at all in normal controls, indicating that the p.P240L mutation arose from a common founder in Koreans.

This is the first documented evidence for the founder mutation hypothesis of p.P240L of *CDH23*. Previous studies on *CDH23* mutations in Caucasian populations did not show clear founder mutations due to the heterogeneous ethnicities of these populations [5, 6, 11, 14]. Instead, most of the *CDH23* variants from different ethnic backgrounds were assumed to be private [26, 27]. Several reports identified founder effects of frequently encountered mutations in specific ethnic populations. Regarding mutations in *GJB2*, several studies reported the frequency of 235delC among East Asians [22], 35delG in the Caucasians [28], and 167delT in Ashkenazi Jews [29], suggesting that these were the result of a founder effect, rather than a mutational hot spot. The most common deafness gene in our cohort, *SLC26A4*, contained the H723R and IVS7-2A>G founder mutations, which are unique to East Asians [30]. The p.A306 mutation of *TMPRSS3* was also recently reported to possess a founder role in Koreans [23].

Most *CDH23* missense mutations localized in the calcium-binding sequences were associated with nonsyndromic hearing loss, which was presumed to be attributable to the increased sensitivity of the cochlear function to calcium-dependent cell adhesion, compared to that of the retinal function [15]. p.P240L is a missense mutation that affects a residue localized in the EC3 domain, with the highly conserved calcium-binding motifs, and therefore does not interfere with calcium binding. Instead, the change from the rigid cyclic side chain of proline to a longer and more flexible leucine residue, results in instability in the structure and function of the protein [18]. To date, the functional defects caused by the p.P240L mutation have not been determined in vitro or in vivo. However, this mutation has been identified only in subjects with nonsyndromic hearing loss (DFNB12) [13, 18, 19]. Furthermore, based solely on a previous audiologic phenotypic study, p.P240L carriers usually show congenital and more severe SNHL than do carriers of other missense mutations in *CDH23* [13]. In accordance with this finding, the four pediatric subjects in this study with p.P240L showed prelingual and severe-to-profound nonsyndromic SNHL with minimal residual hearing at all frequencies.

The p.P240L allele can be classified as a DFNB12 allele, rather than an USH1D allele, based on a previous hypothesis [11] and the phenotype of p.P240L carriers in East Asian populations [13, 18, 19]. The higher prevalence of this DFNB12 allele of p.P240L compared to other *CDH23* mutant alleles implies that in Koreans the nonsyndromic hearing loss (DFNB12) phenotype is more commonly associated with *CDH23* mutations than is the syndromic hearing loss phenotype (USH1D).

The geographic or ethnic spectra of founder mutations vary, and whether the most frequent mutation allele represents a founder mutation and/or a mutational hot spot is often unclear. Some founder mutations have a narrow distribution; for example, due to the practice of consanguineous marriage, the high prevalence of *GJB2* mutations in some Western cultures can be attributed to a single ancestor mutation [31]. On the other hand, the p.A306T mutation of *TMPRSS3* affects a wide ethnic spectrum, encompassing East Asians and Europeans, and hence is predicted to be a mutational hot spot [23, 32, 33]. However, the distribution of several founder alleles, such as the 235delC mutation of *GJB2* and the H723R and IVS7-2A>G mutations of *SLC26A4*, is restricted to East Asians [22, 30]. Only a single cohort of Koreans was investigated in this study, a high prevalence of the p.P240L mutation in East Asians has been reported by others [13, 18, 19, 34], whereas this mutation was rarely reported in other ethnic groups [5, 6, 14]. Therefore, p.P240L is likely a founder mutation exclusive to East Asians. Information on the distinct distribution pattern of the deafness mutations may facilitate determination of the phylogenetic evolution of the deafness genes. Although its value would be limited due to a lack of data on Mongolian or other Asian populations, the different frequency of the p.P240L mutation between East Asian populations and other ethnicities suggests that the p.P240L mutation occurred sometime after the divergence of several ethnic groups from Central or East Asia ~40,000 years ago [35, 36]. Further studies using other genetic polymorphisms, such as single-nucleotide polymorphisms (SNPs) and STR markers adjacent to the p.P240L mutation, would enable a more accurate estimate of the age of this mutation. Further data on the prevalence of *CDH23* mutations and the haplotypes in other ethnic groups would enable tracing of the common ancestor or route of divergence of the *CDH23* mutation. This information would have phylogenetic applications.

## Conclusion

The genetic load of *CDH23* mutations in Korean children with prelingual onset, nonsyndromic, sporadic or autosomal recessive, severe-to-profound SNHL was 3.1 %. p.P240L was the most common *CDH23* mutation. Genotyping of STR markers linked to p.P240L showed that the p.P240L mutation had a strong founder effect in Koreans. This novel finding may facilitate genetic diagnosis of, and research into, *CDH23* mutations, as well as provide information for further phylogenetic studies.

## Additional file

**Additional file 1.** Lists of the genes included in the three targeted panel.
