# Supplementary material for: Strong founder effect of p.P240L in CDH23 in Koreans and its significant contribution to severe-to-profound nonsyndromic hearing loss in a Korean pediatric population
Source: J Transl Med. 2015 Aug 13;13:263. doi: 10.1186/s12967-015-0624-8 (PMC4534105; doi:10.1186/s12967-015-0624-8)
Supplement: Supplementary file 1 — Additional file 1. Lists of the genes included in the three targeted panel. [file 12967_2015_624_MOESM1_ESM.docx]

**Additional file 1** Lists of the genes included in the three targeted panel.

| No. | Gene Symbol | NCBI reference | 80 genes | 129 genes | 204 genes |
| --- | --- | --- | --- | --- | --- |
| 1 | ACTB | NM_001101.3 | O | O | O |
| 2 | ACTG1 | NM_001199954.1 | O | O | O |
| 3 | ALDH1A2 | NM_170697.1 |  |  | O |
| 4 | APAF1 | NM_181869.1 |  |  | O |
| 5 | ATF2 | NM_001880.2 |  |  | O |
| 6 | ATOH1 | NM_007500 |  |  | O |
| 7 | ATP2B2 | [NM_001683.1](http://www.ncbi.nlm.nih.gov/UniGene/clust.cgi?UGID=2547053&TAXID=9541&SEARCH=ATP2B2%20NM) |  |  | O |
| 8 | ATP6V1B1 | NM_001692.3 | O | O | O |
| 9 | ATP6V1B2 | NM_001693.3 |  | O |  |
| 10 | AXIN1 | NM_181050.2 |  |  | O |
| 11 | BCS1L | NM_001079866.1 | O | O | O |
| 12 | BDNF | NM_ 001143809.1 |  |  | O |
| 13 | BMP4 | NM_130850.2 |  |  | O |
| 14 | BMP5 | NM_ 021073 |  |  | O |
| 15 | BSND | NM_057176.2 | O | O | O |
| 16 | CACNG2 | [NM_006078.2](http://www.ncbi.nlm.nih.gov/UniGene/clust.cgi?UGID=2544235&TAXID=9541&SEARCH=CACNG2%20NM) |  |  | O |
| 17 | CAT | NM_001752.1 |  |  | O |
| 18 | CATSPER2 | NM_001282310.1 | O | O | O |
| 19 | CCDC50 | NM_178335.2 | O | O | O |
| 20 | CDH23 | NM_022124.5 | O | O | O |
| 21 | CDKN1B | [NM_004064.2](http://www.ncbi.nlm.nih.gov/UniGene/clust.cgi?UGID=1922177&TAXID=9541&SEARCH=CDKN1B%20NM) |  |  | O |
| 22 | CEACAM16 | NM_001039213.2 |  | O |  |
| 23 | CLDN14 | NM_001146077.1 | O | O | O |
| 24 | CLRN1 | NM_174878.2 |  | O | O |
| 25 | COCH | NM_001135058.1 | O | O | O |
| 26 | COL11A1 | NM_001854.2 |  |  | O |
| 27 | COL11A2 | NM_080680.2 | O | O | O |
| 28 | COL1A1 | NM_007742 |  |  | O |
| 29 | COL1A2 | NM_000089.3 |  |  | O |
| 30 | COL2A1 | NM_001844.4 |  |  | O |
| 31 | COL4A3 | NM_000091.4 |  |  | O |
| 32 | COL9A2 | NM_001852.3 |  | O |  |
| 33 | COL9A3 | NM_001853.3 | O | O | O |
| 34 | CRYM | NM_001888.3 | O | O | O |
| 35 | CSF1 | NM_007778.4 |  |  | O |
| 36 | DFNA5 | NM_004403.2 | O | O | O |
| 37 | DFNB31 | NM_001173425.1 | O | O | O |
| 38 | DFNB59 | NM_001042702.3 |  | O | O |
| 39 | DIAPH1 | NM_005219.4 | O | O | O |
| 40 | DLX1 | NM_010053.1 |  |  | O |
| 41 | DLX2 | NM_010054.2 |  |  | O |
| 42 | DLX5 | NM_198854.1 |  |  | O |
| 43 | DMD | NM_007868.5 |  |  | O |
| 44 | DSPP | NM_014208.3 | O | O | O |
| 45 | ECE1 | NM_001397.2 |  | O | O |
| 46 | EDN1 | NM_001955.4 |  |  | O |
| 47 | EDN3 | NM_007903.3 |  |  | O |
| 48 | EDNRA | NM_001957.3 |  | O | O |
| 49 | EDNRB | NM_000115.3 |  | O | O |
| 50 | ERCC2 | NM_000400.3 | O | O | O |
| 51 | ERCC3 | NM_000122.1 | O | O | O |
| 52 | ESPN | NM_031475.2 | O | O | O |
| 53 | ESRRB | NM_004452.3 | O | O | O |
| 54 | EYA1 | NM_000503.4 |  |  | O |
| 55 | EYA4 | NM_004100.4 | O | O | O |
| 56 | F2 | NM_000506.3 |  |  | O |
| 57 | F5 | NM_000130.4 |  |  | O |
| 58 | FAS | NM_000043.4 |  | O | O |
| 59 | FGF3 | NM_005247.2 | O | O | O |
| 60 | FGF8 | [NM_001166363.1](http://www.ncbi.nlm.nih.gov/UniGene/seq.cgi?ORG=Mm&SID=53249940) |  |  | O |
| 61 | FGFR2 | NM_000141.4 |  |  | O |
| 62 | FGFR3 | NM_000142.4 |  | O | O |
| 63 | FOXI1 | NM_012188.4 |  | O | O |
| 64 | GATA3 | NM_001002295.1 | O | O | O |
| 65 | GIPC3 | NM_133261.2 |  | O |  |
| 66 | GJA1 | NM_000165.3 | O | O | O |
| 67 | GJA4 | NM_002060.2 |  |  | O |
| 68 | GJB1 | NM_000166.5 | O | O | O |
| 69 | GJB2 | NM_004004.5 | O | O | O |
| 70 | GJB3 | NM_001005752.1 | O | O | O |
| 71 | GJB4 | NM_153212.2 | O | O | O |
| 72 | GJB5 | NM_010291.3 |  |  | O |
| 73 | GJB6 | NM_001110219.2 | O | O | O |
| 74 | GLI2 | NM_001081125.1 |  |  | O |
| 75 | GLI3 | NM_008130.2 |  |  | O |
| 76 | GPR98 | NM_032119.3 |  | O | O |
| 77 | GPSM2 | NM_013296.4 |  | O |  |
| 78 | GRHL2 | NM_024915.3 | O | O | O |
| 79 | GRID1 | NM_017551.2 |  |  | O |
| 80 | GRXCR1 | NM_001080476.2 |  | O |  |
| 81 | GSC | NM_131017.1 |  |  | O |
| 82 | GSTP1 | NM_000852.3 | O | O | O |
| 83 | HAL | NM_002108.3 |  | O |  |
| 84 | HAPLN1 | NM_001884.3 |  |  | O |
| 85 | HGF | NM_000601.4 |  | O |  |
| 86 | HMX2 | NM_145998.3 |  |  | O |
| 87 | HMX3 | NM_008257.3 |  |  | O |
| 88 | HOXA1 | NM_010449.4 |  |  | O |
| 89 | HOXA2 | NM_010451.1 |  |  | O |
| 90 | HOXB1 | NM_008266.5 |  |  | O |
| 91 | HOXB2 | NM_134032.2 |  |  | O |
| 92 | HSPG2 | NM_005529.5 |  |  | O |
| 93 | ILDR1 | NM_001199799.1 |  | O |  |
| 94 | ITGA1 | NM_001033228.3 |  |  | O |
| 95 | ITGA3 | NM_002204.2 |  |  | O |
| 96 | ITGA8 | NM_001001309.2 |  |  | O |
| 97 | JAG1 | NM_000214.2 | O | O | O |
| 98 | JAG2 | NM_010588.2 |  |  | O |
| 99 | KCNE1 | NM_000219.4 | O | O | O |
| 100 | KCNJ10 | NM_002241.4 | O | O | O |
| 101 | KCNQ1 | NM_000218.2 |  | O |  |
| 102 | KCNQ4 | NM_004700.3 | O | O | O |
| 103 | KIAA1199 | NM_018689.1 |  | O |  |
| 104 | KIT | NM_001122733.1 |  |  | O |
| 105 | KRML | NM_005461 |  |  | O |
| 106 | LAMA2 | NM_008481.2 |  |  | O |
| 107 | LHFPL5 | NM_182548.3 | O | O | O |
| 108 | LHX3 | NM_014564.3 | O | O | O |
| 109 | LMX1A | NM_033652.5 |  |  | O |
| 110 | LOXHD1 | NM_144612.6 |  | O |  |
| 111 | LRTOMT | NM_001145309.3 | O | O | O |
| 112 | MARVELD2 | NM_001038603.2 |  | O | O |
| 113 | MBP | NM_001025251.2 |  |  | O |
| 114 | mir182 | NR_029614.1 |  | O |  |
| 115 | mir183 | NR_029615.1 |  | O |  |
| 116 | mir96 | NR_029512.1 |  | O |  |
| 117 | MITF | NM_198159.2 |  | O | O |
| 118 | MOS | NM_020021.2 |  |  | O |
| 119 | MPV17 | NM_002437.4 |  |  | O |
| 120 | MPZ | NM_000530.6 |  |  | O |
| 121 | MSRB3 | NM_198080.3 |  | O |  |
| 122 | MSX1 | NM_010835.2 |  |  | O |
| 123 | MSX2 | NM_013601.2 |  |  | O |
| 124 | MTAP | NM_002451.3 | O | O | O |
| 125 | MTHFR | NM_005957.4 |  |  | O |
| 126 | MT-RNR1 | NC_012920.1 |  |  | O |
| 127 | MT-TD | NC_012920. |  | O |  |
| 128 | MT-TH | NC_012920.1 |  | O |  |
| 129 | MT-TI | NC_012920.1 |  | O |  |
| 130 | MT-TK | NC_012920.1 |  | O |  |
| 131 | MT-TL1 (=TRNL1) | NC_012920.1 |  | O | O |
| 132 | MT-L2 (=MTTS1) | NC_012920.1 |  | O | O |
| 133 | MT-TM | NC_012920.1 |  | O |  |
| 134 | MT-TQ | NC_012920.1 |  | O |  |
| 135 | MT-TS1 | NC_012920.1 |  | O |  |
| 136 | MT-TS2 | NC_012920.1 |  | O |  |
| 137 | MYH14 | NM_001145809.1 | O | O | O |
| 138 | MYH9 | NM_002473.4 | O | O | O |
| 139 | MYO15A | NM_016239.3 | O | O | O |
| 140 | MYO1A | NM_001256041.1 | O | O | O |
| 141 | MYO1C | NM_001080779.1 | O | O | O |
| 142 | MYO1F | NM_012335.3 | O | O | O |
| 143 | MYO3A | NM_017433.4 | O | O | O |
| 144 | MYO6 | NM_004999.3 | O | O | O |
| 145 | MYO7A | NM_000260.3 | O | O | O |
| 146 | NDP | NM_000266.3 |  | O | O |
| 147 | NEUROG1 | NM_131041.1 |  |  | O |
| 148 | NF2 | NM_000268.3 |  |  | O |
| 149 | NR2F1 | XM_005272070.1 | O | O | O |
| 150 | NTF3 | NM_001164034.1 |  |  | O |
| 151 | NTN1 | NM_008744.2 |  |  | O |
| 152 | OTOA | NM_144672.3 | O | O | O |
| 153 | OTOF | NM_194248.2 | O | O | O |
| 154 | OTOR | NM_020157.3 | O | O | O |
| 155 | OTX1 | NM_011023.3 |  |  | O |
| 156 | OTX2 | NM_144841.3 |  |  | O |
| 157 | P73 | NM_005427.3 |  |  | O |
| 158 | P2RX2 | NM_170683.3 |  | O |  |
| 159 | PAX2 | NM_181457.3 |  | O | O |
| 160 | PAX3 | NM_008781.4 | O |  | O |
| 161 | PAX5 | NM_008782.2 |  |  | O |
| 162 | PAX9 | NM_011041.2 |  |  | O |
| 163 | PCDH15 | NM_001142763.1 | O | O | O |
| 164 | PDZD7 | NM_001195263.1 | O | O | O |
| 165 | PEJVAKIN | NM_001042702.3 | O |  | O |
| 166 | PHEX | NM_000444.4 |  |  | O |
| 167 | PLAT | NM_000930.3 |  |  | O |
| 168 | PLDN | NM_012388.2 |  |  | O |
| 169 | PMP22 | NM_000304.3 | O | O | O |
| 170 | PON1 | NM_000446.5 |  |  | O |
| 171 | PON2 | NM_000305.1 |  |  | O |
| 172 | POU1F1 | NM_000306.2 |  |  | O |
| 173 | POU3F4 | NM_000307.4 | O | O | O |
| 174 | POU4F1 | NM_011143.4 |  |  | O |
| 175 | POU4F3 | NM_002700.2 | O | O | O |
| 176 | PRODH | NM_016335.4 |  |  | O |
| 177 | PROP1 | NM_006261.4 |  |  | O |
| 178 | PRPS1 | NM_002764.3 |  | O |  |
| 179 | PRRX1 | NM_006902.3 |  |  | O |
| 180 | PRRX2 | NM_016307.3 |  |  | O |
| 181 | PTPRQ | NM_001145026.1 |  | O |  |
| 182 | RARA | NM_000964.3 |  |  | O |
| 183 | RARB | NM_000965.3 |  |  | O |
| 184 | RARG | NM_000966.5 |  |  | O |
| 185 | RDX | NM_001260492.1 | O | O | O |
| 186 | RELN | NM_005045.3 |  |  | O |
| 187 | RNR1 | NM_127748.3 |  |  | O |
| 188 | RORA | NM_134261.2 |  |  | O |
| 189 | SERPINE1 | NM_000602.4 |  |  | O |
| 190 | SERPINB6 | NM_004568.5 |  | O |  |
| 191 | SIX1 | NM_005982.3 |  | O |  |
| 192 | SIX5 | NM_175875.4 |  | O |  |
| 193 | SLC12A2 | NM_001046.2 |  |  | O |
| 194 | SLC17A8 | NM_139319.2 | O | O | O |
| 195 | SLC26A4 | NM_000441.1 | O | O | O |
| 196 | SLC26A5 | NM_198999.2 | O | O | O |
| 197 | SLC30A4 | NM_013309.4 |  |  | O |
| 198 | SLC4A11 | NM_001174090.1 | O | O | O |
| 199 | SLC9A1 | NM_003047.4 |  |  | O |
| 200 | SMPX | NM_014332.2 |  | O |  |
| 201 | SNAI2 | NM_003068.4 |  | O |  |
| 202 | SNAP25 | NM_003081.3 |  |  | O |
| 203 | SOD1 | NM_000454.4 |  |  | O |
| 204 | SOD2 | [NM_000636.2](http://www.ncbi.nlm.nih.gov/UniGene/seq.cgi?ORG=Hs&SID=3218924) |  |  | O |
| 205 | SOX10 | NM_006941.3 |  |  | O |
| 206 | SOX2 | NM_003106.3 | O | O | O |
| 207 | SPINK5 | NM_001127698.1 | O | O | O |
| 208 | STRC | NM_153700.2 | O | O | O |
| 209 | TBL1X | NM_005647.3 | O | O | O |
| 210 | TCF21 | NM_198392.2 | O | O | O |
| 211 | TECTA | NM_005422.2 | O | O | O |
| 212 | TFAP2A | NM_003220.2 |  |  | O |
| 213 | TFCP2 | NM_005653.4 |  | O |  |
| 214 | TFCP2L3 | NM_024915 | O |  | O |
| 215 | TGFA | NM_003236.3 |  |  | O |
| 216 | TGFB2 | NM_001135599.2 |  |  | O |
| 217 | THRA | NM_199334.3 |  |  | O |
| 218 | THRB | NM_000461.4 |  |  | O |
| 219 | TIMM8A | NM_004085.3 | O | O | O |
| 220 | TJP2 | NM_004817.3 |  | O |  |
| 221 | TMC1 | NM_138691.2 | O | O | O |
| 222 | TMIE | NM_147196.2 | O | O | O |
| 223 | TMPRSS3 | NM_024022.2 | O | O | O |
| 224 | TMPRSS5 | NM_030770.2 | O | O | O |
| 225 | TNC | NM_002160.3 |  |  | O |
| 226 | TPRN | NM_001128228.2 |  | O |  |
| 227 | TRIC | NM_001038603.2 | O |  | O |
| 228 | TRIOBP | NM_001039141.2 | O | O | O |
| 229 | TRMU | NM_028063.2 |  |  | O |
| 230 | TSHR | NM_000369.2 |  |  | O |
| 231 | TUB | NM_003320.4 |  |  | O |
| 232 | TYMP | NM_001953.4 |  |  | O |
| 233 | TYR | NM_000372.4 |  |  | O |
| 234 | TYRP1 | NM_000550.2 |  |  | O |
| 235 | UCN | NM_003353.2 |  |  | O |
| 236 | USH1C | NM_153676.3 | O | O | O |
| 237 | USH1G | NM_173477.3 |  | O | O |
| 238 | USH2A | NM_206933.2 |  | O | O |
| 239 | WFS1 | NM_006005.3 | O | O | O |
